# Case Report: Challenges in the diagnosis of pseudohypoaldosteronism: insights from a resource-limited setting

**DOI:** 10.3389/fendo.2026.1708536

**Published:** 2026-05-21

**Authors:** Salwa A. Musa, Nifal S. Ariss, Samar S. Hassan, Omer O. Babiker, Ghassan F. Mohamedsalih, Asmahan T. Abdalla, Mohamed A. Abdullah

**Affiliations:** 1 Department of Paediatric Endocrinology and Diabetes, Gaafar Ibn Auf Paediatric Tertiary Hospital, Khartoum, Sudan; 2 Department of Paediatrics and Child Health, Faculty of Medicine, Al-Neelain University, Khartoum, Sudan; 3 Pediatric Department, The Medical City for Military and Security Services, Muscat, Oman; 4 Department of Paediatrics, Faculty of Medicine and Health Sciences, Omdurman Islamic University, Omdurman, Sudan; 5 Division of Endocrinology, Department of Paediatric Medicine, Sidra Medicine, Doha, Qatar; 6 Department of Pediatrics and Child Health, Faculty of Medicine, International University of Africa, Khartoum, Sudan; 7 Department of Pediatrics and Child Health, Faculty of Medicine, University of Khartoum, Khartoum, Sudan

**Keywords:** CAH, PHA type 1, pseudohypoaldosteronism, resource-limited setting, Sudan

## Abstract

**Background:**

Pseudo-hypoaldosteronism type 1 (PHA1) is a hereditary condition characterized by end-organ resistance to aldosterone, resulting in electrolyte imbalance, metabolic acidosis, and hyperaldosteronism. It is inherited in an autosomal dominant renal or an autosomal recessive systemic form. Here, we report a case series of PHA1, highlighting its challenging diagnosis and underscoring the complexities involved in reaching an accurate diagnosis in a resource-limited setting.

**Case report:**

Seven patients from seven unrelated Sudanese families were admitted to pediatric endocrinology units at Gaffer Ibn Auf (GIA) Children’s Teaching Hospital in Khartoum, Sudan, between 2010 and 2023, with an initial suspected diagnosis of gastroenteritis or Congenital adrenal hyperplasia (CAH). The age of presentation varied significantly between patients (4–120 days). Failure to thrive and dehydration were the most common presenting symptoms, and one patient was initially misdiagnosed due to prominent respiratory symptoms. Consanguinity was reported in 4 families, and three patients had a family history of neonatal fatalities with a similar presentation. Initial electrolyte abnormalities showed low serum sodium, elevated potassium, and metabolic acidosis, which initially pointed to CAH diagnosis. However, normal cortisol and Adrenocorticotropic hormone (ACTH) levels indicated the need to consider alternative diagnoses, and high aldosterone and renin levels suggested a PHA1 diagnosis confirmed by genetic testing in three patients that revealed mutations in SCNNA, B, and G subunits.

**Conclusion:**

The diagnosis of PHA1 is challenging in Sudan, where the clinical and biochemical features can mimic other common conditions contributing to its delayed diagnosis. Failure to respond to initial glucocorticoid (GC) therapy prompted a consideration of other alternative diagnoses, including PHA1, especially in settings where access to advanced facilities for diagnostic confirmation is limited.

## Introduction

Pseudohypoaldosteronism type 1 (PHA1) is a rare hereditary mineralocorticoid resistance disease due to a malfunction in the aldosterone intracellular signaling system (1). The condition presents predominantly in the neonatal period and early infancy with vomiting, dehydration, salt wasting, and failure to thrive, mostly within the first 2 weeks to 6 months of life (2). It is classified into two main types based on clinical and genetic criteria: the more common, less severe autosomal dominant (AD) renal type and the more severe, systemic, autosomal recessive (AR) type (3, 4). The former (AD type) is caused by an inactivating mutation in the mineralocorticoid receptor encoded by the NR3C2 gene, and symptoms typically resolve with age and require lower sodium requirements in infancy and childhood (5). The latter is caused by an inactivating mutation in any of three subunits (alpha, beta, gamma) of the amiloride-sensitive epithelial sodium channel (ENaC) encoded by the SCNN1A, B, G genes. ENAC is found in the apical membrane of salt-reabsorbing tight epithelia in the distal nephron, distal colon, salivary and sweat glands, and lung, where it serves as a rate-limiting stage in the transfer of Na+ ions. Thus, this type presents more severely with dehydration, life-threatening electrolyte disturbances, pulmonary manifestations such as recurrent respiratory tract infection, cystic fibrosis-like symptoms and elevated sweat electrolytes indicating multiorgan involvement requiring high-dose sodium supplementation (6, 7).

The classic biochemical findings of PHA1 include hyponatremia, hyperkalemia, metabolic acidosis, elevated renin, and extremely high aldosterone, with normal cortisol and 17-hydroxyprogesterone levels (17OHP), which distinguish PHA from adrenal insufficiency seen in congenital adrenal hyperplasia (4, 8). The differential diagnosis includes CAH and hypoaldosteronism, which adds to the diagnostic complexity, particularly in settings where access to hormonal assays or genetic testing is limited. Sodium chloride supplements and cation-exchange resins are the cornerstones of PHA-1 therapy, whereas oral NaHCO3 can be used to treat metabolic acidosis (9). Here, we present seven Sudanese patients with PHA1 seen in a single center in Sudan between 2010 and 2023, describing their clinical phenotype and challenging diagnosis in the context of nonspecific presentation and similarity of their presentation to other prevalent conditions in the area, such as CAH (10).

## Cases

### Case 1

A four-day-old female born to non-consanguineous parents presented with vomiting. There is a family history of a male sibling’s death in the neonatal period with a similar presentation. She was dehydrated at presentation with normal female genitalia, and systemic examination was unremarkable. Electrolytes upon workup revealed low Na of (127 mmol/l), high Potassium (7 mmol/l), indicating salt loss in addition to metabolic acidosis (HCO3 13.3 mmol/l). Given these electrolyte abnormalities, and although she is a female, the initial cortisol and ACTH levels were taken at the primary hospital to exclude CAH, and dehydration was corrected, along with initiation of GC, with no response. The result revealed normal cortisol (1144 nmol/l) and ACTH (13.7 pmol/l), followed by 17OHP after 3 weeks, which came back normal (9nmol/l); all warranted further workup. Renin and Aldosterone were reported as high, supporting PHA1 diagnosis. The patient was started on potassium-binding resin and sodium of 15 mmol/kg/day. Genetic testing was not affordable for this child, but she showed clinical improvement on follow-up.

### Case 2

A 90-day-old male infant presented to our secondary hospital with vomiting, dehydration, and failure to thrive, with no other relevant family history. He has normal male genitalia, and no skin hyperpigmentation was noted. After initial resuscitation, laboratory evaluation showed hyponatremia (121 mmol/L) and hyperkalemia (6.5 mmol/L). Given these biochemical abnormalities, a diagnosis of CAH was suspected, and glucocorticoid therapy was initiated, but did not produce an adequate response. This diagnosis was re-evaluated due to the normal cortisol (573 nmol/l) and ACTH (28pg/ml) in addition to the 17-OHP level of (6.9 nmol/L). The diagnosis of PHA1 was established, and the patient continued on potassium-binding resin, with a sodium supplementation requirement of 10 mmol/kg/day.

### Case 3

A three-month-old male infant born to consanguineous parents presented with failure to thrive for two months, accompanied by several episodes of vomiting and dehydration that required multiple hospitalizations. The parents are consanguineous, but there is no family history of similar conditions or infant death. Physical examination showed normal male genitalia with no skin abnormalities. Laboratory evaluation at presentation revealed severe hyponatremia (118 mmol/L) and hyperkalemia (6 mmol/L). The patient was initially stabilized and treated as having CAH with glucocorticoids (GC) and mineralocorticoids (MC), but there was no response in terms of acidosis or electrolyte correction. The cortisol level was borderline at 253 nmol/l, and the 17-OHP level was mildly elevated at 20 nmol/L (<12.1-18.1), raising the suspicion of the CAH diagnosis. However, synacthen was performed and confirmed a normal cortisol response. The patient’s condition deteriorated despite adequate GC and MC therapy, prompting a reassessment of the diagnosis. Further evaluation revealed aldosterone at 762 ng/dL and renin levels greater than 128, both significantly elevated. Genetic testing was not performed due to financial constraints, but the response to sodium supplementation at 11 mmol/kg/day and potassium-binding resin supported a diagnosis of PHA1. The patient continued on this management and showed a good response on follow-up, but unfortunately died at the age of 5.5 years due to an unknown event.

### Case 4

A 34-day-old female infant, born to consanguineous parents, presented with vomiting and failure to thrive for a 3-week duration. There was no relevant family history or neonatal death. A hyperpigmented papular skin rash was noted on skin examination. Laboratory evaluation revealed hyponatremia (127 mmol/l), hyperkalemia (8 mmol/l), and metabolic acidosis with a bicarbonate of 9.7 mmol/l. CAH was suspected, and the patient was started on GC and MC pending the 17-OHP level, which was normal (9.7 nmol/l), with reported normal ACTH and cortisol levels, suggesting another diagnosis. PHA1 was suspected, supported by a high aldosterone level of (>100 ng/dl), and high renin of (133.5 IU/ml). Genetic testing identified a homozygous start-loss mutation (c.-6delinsATC) in the SCNN1G gene. Sodium supplementation was initiated at a dose of 5 mmol/kg/day with Calcium-resin. She is on regular follow-up and is currently 5 years and 3 months old, with higher sodium supplementation of 17 mmol/day and calcium resin of 7g/day.

### Case 5

A 5-day-old male infant presented with refusal of feeding and dehydration for 2 days duration. He is a product of non-consanguineous parents with a positive family history of similar presentation and infant death at 14 days of age. Miliaria rubra (a hyperpigmented papular skin rash) was noted on skin examination. Initial laboratory results showed hyponatremia (125 mmol/l), hyperkalemia (7 mmol/l), and metabolic acidosis with a bicarbonate level of 9 mmol/l. Initial suspicion of CAH was made, given the sex and family history, at which immediate GC and MC were commenced. The normal 17-OHP (0.2 nmol/l) level warranted further evaluation. The cortisol level was normal (556 nmol/l), but ACTH was not available at that time. The renin was not available, yet the high aldosterone level of 307 ng/dl) supported PHA1 diagnosis. Genetic testing was not performed. The patient required sodium supplementation of 19 mmol/kg/day and potassium resin. The patient is currently 7 months old with adequate growth parameters.

### Case 6

A 10-day-old male infant born to consanguineous parents presented with refusal of feeding for 3 days. A significant family history of infant deaths was noted, with two siblings dying at the ages of 13 and 14 days. He was dehydrated at presentation, but no hyperpigmentation was observed. Laboratory evaluation revealed hyponatremia (122 mmol/l), hyperkalemia (8.3 mmol/l), and a bicarbonate of 11 mmol/l. Due to a significant history of male deaths along with these electrolyte abnormalities, he was started immediately on GC and MC after initial resuscitation. The result of cortisol (635 nmol/l) was normal, along with a normal 17-OHP level (10 nmol/l), highlighting a non-CAH diagnosis. Renin and aldosterone were reported as highly supported PHA1 diagnosis, which was later confirmed by genetic testing that revealed the presence of homozygous mutations in the SCNN1A gene, including a missense variant (c.517G>A) and a frameshift mutation (c.1343del). Sodium requirement was 16 mmol/kg/day, and the patient is currently 2 years of age and on regular follow-up.

### Case 7

A 14-day-old male infant born to consanguineous parents presented with fever and increased work of breathing. He was born at term to consanguineous parents, with no family history of neonatal deaths. He was initially managed as having pneumonia and showed no clinical response. Since then, the patient has had repeated lung infections, all of which were treated with intravenous antibiotics and bronchodilators with no significant improvement. At 7 months of age, he developed vomiting, diarrhea, and hyponatremic dehydration and potassium was found to be high. Examination at 7 months of age showed a hyperpigmented papular skin rash between the digits, in the axilla, and over the trunk. Initial Laboratory evaluation revealed hyponatremia (109 mmol/l), hyperkalemia (7.9 mmol/l), and a bicarbonate level of 10 mmol/l. Patient was treated adequately with i.v fluids along with i.v hydrocortisone after a sample of 17-OHP was taken, revealing a normal result (1.8 nmol/l). The cortisol level was (538nmol/l), and ACTH (18pg/ml), both were normal, as well as aldosterone and renin levels. Homozygous missense variant c.1636G>AP. (Asp546Asn) in the SCNN1B gene was found on genetic testing. The patient required sodium supplementation of 17 mmol/kg/day and potassium resin, at which he showed clinical improvement, but he lost follow-up recently due to the ongoing war. All seven cases with their features at initial presentation have been described in (**Table 1**).

**Table 1:** Clinical features of PHA1 cases at presentation (n=7).

Patients	P1	P2	P3	P4	P5	P6	P7
Age at initial presentation	4 days	90 days	120 days	34 days	5 days	10 days	14 days
Sex	Female	Male	Male	Female	Male	Male	Male
Consanguinity	No	No	Yes	Yes	No	Yes	Yes
Presenting symptoms	Vomiting	Vomiting + FTT	FTT	Vomiting + FTT	Refusal of feeding	Refusal of feeding	Fever + increased breathing effort.
F.H. of a similar presentation	Yes	No	No	No	Yes	Yes	No
F.H. of early infant death	Yes Male sibling	No	No	No	Yes Sibling at 14 days	Yes 2 siblings at 13, 14 days	No
Skin abnormalities	No	No	No	Yes	Yes	No	No
Initial S. Na (mmol/L)	127	121	118	127	125	122	109
Initial S. K (mmol/L)	7	6.5	6	8	7	8.3	7.9
Initial S. Hco3 (mmol/L)	13.3	NA	NA	9.7	9	11	10
17 OHP N = (<12.1-18.1 nmol/l)	9	6.9	20	9.7	0.2	10	1.8
ACTH N = (7.2-63.3 pg/ml)	13.7	28	12	Normal	NA	NA	18
Cortisol N= (171-536 nmol/L)	1144	537	253	Normal	556	635	538
Aldosterone (ng/dl)	NA	NA	762	>100	307	NA	100
Renin	NA	NA	>128(4.7-42ng/ml)	133.5 iu/ml (4.4-46.1)	NA	NA	4.2 (0.1–0.75 pmol/L)
Sweat test	NA	NA	NA	NA	NA	NA	Chloride = 112.66 mmol/L (10–65) Sodium = 179.27 mmol/L (10–70)
Genetic testing	NA	NA	NA	Homozygous Start-lost c.-6_6delinsATC in SCNN1G gene	NA	Homozygous Missense variant c.517G>A in the SCNN1A and Frameshift c.1343del in the SCNN1A gene	Homozygous missense variant c.1636G>AP. (Asp546Asn) in the SCNN1B gene
Sodium requirements	15 mmol/kg/day	10 mmol/kg/day	11 mmol/kg	17 mmol/kg/day	19 mmol/kg/day	16 mmol/kg/day	17 mmol/kg/day

(FTT, Failure to thrive; NA, not available).

## Discussion

PHA1 is a rare clinical disorder characterized by mineralocorticoid resistance due to a lack of response to aldosterone in the target cells. This results in hyponatremia, hyperkalemia, and metabolic acidosis, typically presenting in the neonatal period and early infancy with vomiting, dehydration, and failure to thrive. It is characterized by elevated plasma aldosterone and plasma renin activity, in contrast to PHA type 2, where patients present with hypertension, hyperkalemia, metabolic acidosis, normal plasma aldosterone, and decreased plasma renin activity (11, 12).

Here, we are reporting seven patients (5 males and 2 females) from seven unrelated Sudanese families, who have been reviewed at the pediatric endocrinology units at Gaafar Ibn Auf (GIA) hospital, the main pediatric tertiary center in Khartoum, Sudan, with confirmed PHA1 diagnosis. This series highlights the existing gap in published records regarding PHA1 in Sub-Saharan Africa and emphasizes the importance of clinical recognition of the condition in diverse healthcare environments, particularly in low-resource settings (13, 14).

Consanguinity was reported in specific cases of systemic PHA, suggesting an AR mode of inheritance (4, 13, 15). It was identified in 4 families in this series, while three families reported having one or two siblings who died with a similar presentation at different ages. Consanguinity in the Sudanese population might reach as high as 80%, which significantly increases the risk of AR disorders (10). A similar presentation among other siblings and reported deaths emphasizes the diagnostic overlap with other conditions, leading to delayed diagnosis.

While PHA1 typically presents early in life, with the (AR) systemic form often appearing within the first week of life, while the (AD) renal form within the first two months, our cohort demonstrated significant clinical variability with an age at presentation ranging from as early as 4 days to as late as 120 days. Notably, two patients with genetically confirmed AR systemic disease (P4 and P7) presented well beyond the typical first-week window. Furthermore, two other patients (P2 and P3) who lacked a genetic diagnosis but exhibited a clinical phenotype suggestive of the milder AD form experienced a delayed diagnosis beyond three months compared to other series (2, 8).

Symptoms at presentation included poor feeding, vomiting, dehydration, refusal of feeding, and failure to thrive, and patients were commonly initially diagnosed with gastroenteritis before referral. Weight loss and failure to thrive emerged as a prominent presenting feature across multiple studies, including ours, where patients were commonly initially diagnosed with gastroenteritis before referral (4, 15). The severity of presentation varied by PHA 1 subtypes, with AD renal that typically shows a milder course with clinical expression varying from asymptomatic to moderate in affected family members. This is in contrast to AR systemic forms, where patients presented with life-threatening, severe hyperkalemia and hyponatremia (2, 4, 8, 15). In addition, patients with this type have reduced sodium-dependent fluid absorption in the lungs, leading to respiratory symptoms and a cystic fibrosis-like phenotype, with a variable clinical course depending on both electrolyte imbalance and respiratory symptoms (16, 17). This could lead to masking the appropriate diagnosis, leading to further delay in diagnosis, as in (P7), where he had a challenging presentation of recurrent admissions with respiratory symptoms that had been managed as pneumonia and received intravenous antibiotics and bronchodilators, which all contributed to masking and delaying his final diagnosis. This deviation highlights the nonspecific nature of early symptoms, which can lead to diagnostic challenges, and underscores how PHA1 can be easily masked by more frequent pediatric presentations early in life. This patient had a positive sweat test, indicating an elevated sweat chloride level, with later confirmation of PHA1 diagnosis by genetic testing.

The combination of clinical features and a family history suggestive of neonatal deaths, often seen in the context of high consanguinity, initially pointed toward CAH as a top differential diagnosis. These clinical features are frequently encountered in the salt-wasting forms of CAH, where male neonates typically exhibit salt loss and/or adrenal crises within the first few weeks of life. The lack of genital virilization in males makes it very challenging to differentiate CAH from other adrenal disorders like PHA1. In contrast, while atypical genitalia in females usually facilitates a more rapid diagnosis, this clinical sign does not always guarantee a straightforward path; notably, two females in this series underwent extensive workup at their initial facilities to exclude CAH before PHA1 was considered. This suggests that even when physical signs are clear, the rarity of PHA1 can lead to a narrow focus on more common adrenal disorders and contribute to diagnostic delays. Ultimately, in this series, the clinical diagnosis of CAH was challenged by the persistence of electrolyte abnormalities despite standard replacement GC and MC therapy, necessitating further evaluation of the mineralocorticoid resistance pathway (18). We emphasized that in the absence of advanced laboratory facilities, achieving a correct diagnosis relies heavily on high clinical suspicion. For an infant presenting with refractory hyperkalemia, the failure to respond to initial steroid therapy is a critical diagnostic milestone. In a resource-constrained context, this clinical non-response should immediately raise suspicion of an alternative diagnosis, such as PHA1, especially since male neonates lack the distinctive genital features often seen in female cases of CAH.

Physical signs of PHA1 included severe dehydration, bullous dermatitis, and chronic xerosis in some patients with systemic PHA (19). Miliria rubra-like rash was detected in 3 patients in this series at presentation. It is hypothesized that during a salt-losing crisis, the eccrine ducts are directly affected by the maximum sodium chloride concentrations achieved in the sweat. The fact that three of our patients manifested this skin condition specifically during their salt-wasting episodes suggests that such a sign could serve as a clinical warning signal that may allow for the early detection of an impending crisis, enabling early prevention and timely intervention (19–22).

All patients across different studies shared a similar triad of specific, defined biochemical abnormalities of hyponatremia (sodium <135 mmol/L), hyperkalemia (serum K+ >5.0 mmol/L), and metabolic acidosis (bicarbonate <20 mmol/L) (12, 23). This was similar in all cases in this series during initial evaluation with a serum sodium (range109-127mmol/l), potassium (range 6–8.3mmol/l), and serum bicarbonate (range 9-13mmol/L) during initial evaluation. Urine electrolytes were assessed in two patients (P4, P6), showing low urinary potassium and high urinary sodium levels. Urine electrolytes are difficult to evaluate in most of our patients except for two patients: P (6), who had low urine K levels, and P (4), who had a high urine Na/K ratio, demonstrating a lack of mineralocorticoid receptors and salt loss despite hyperaldosteronism. Urine culture and abdominal ultrasound helped rule out any obstruction or abnormality of the urinary system and excluded the possibility of secondary PHA, which can cause transient aldosterone resistance. High blood aldosterone levels and plasma renin activity despite electrolyte abnormalities are the hallmark hormonal pattern in all patients compatible with PHA1 diagnosis (23). Simultaneous measurements of plasma cortisol and ACTH for suspected patients, in addition to 17OHP if available, were found to reduce potential misinterpretation of clinical data, particularly in male newborns and in countries where CAH is not uncommon. However, 17OHP was found to be borderline high in one patient (P3) during his initial workup. It was proposed that a high 17OHP was found to be high during severe illness, which does not necessarily indicate CAH (24, 25).

The differentiation between renal and systemic types of PHA1 is crucial to distinguish systemic types of patients who are more prone to decompensation. However, that was only possible in 3 cases due to financial limitations and the high cost of genetic testing in a low-resource setup like ours. This revealed two mutation variants in SCNN1A (homozygous missense variant c.517G>A in SCNN1A and frameshift c.1343del in the SCNN1A gene) in (P6) and a homozygous missense variant c.1636G>AP. (Asp546Asn) in the SCNN1B gene in (P7), both have been reported previously in different series (8)’ (19)’ (20). Missense mutant patients tend to have a milder disease course, yet patient P (6) had an initial severe presentation and continued to require high salt supplements (26). This highlights the phenotypic variability in PHA1 patients, possibly due to the large distribution of the ENaC subunit (8). A homozygous start-loss c.-6 6delinsATC in the SCNN1G gene was found in (P4), where only a few cases have been documented in the literature reporting (2, 8, 15). The identification of mutations in the SCNN1A, SCNN1B, and SCNN1G genes further confirmed the final diagnosis and provided significant support for family counselling and future planning. All patients received initial stabilization and rehydration, oral salts for hyponatremia, and the management of hyperkalemia and metabolic acidosis with potassium-binding resin. However, those with systemic form on genetic testing required higher salt intake (up to 17mmol/day) and intensive fluid and electrolyte management compared to others (P4,6,7). In the absence of genetic testing, this response pattern might provide additional classification criteria that help in the differentiation between two distinct types of PHA1, with renal PHA1 responding well to sodium supplementation and requirements often decreasing with age, while the systemic form requires higher salt intake with more severe courses (4, 8, 15). However, this could be difficult when some systemic PHA1 might run a milder course with low salt requirements, as in P (4). This can be explained by increased Na+ reabsorption from renal tubules in systemic PHA1 because of increased Na–Cl cotransporter protein, which increases with age, similarly reported in some series, compared to high requirements reported in others (27, 28).

Systemic PHA1 presents a significantly more severe clinical course compared to the renal variant, with high morbidity and mortality primarily driven by life-threatening hyperkalemia and acute dehydration. While severe neurological impairment has been reported as a sequela of hyperkalemia-induced cardiac arrest, one patient in our cohort (P4) has recently presented with new-onset abnormal movements requiring further investigation. Respiratory complications were managed by bronchodilators, chest physiotherapy, and targeted antibiotics when required.

Long-term follow-up remains difficult and is hindered by logistical barriers, leading to missed appointments; thus, we have increasingly utilized telemedicine to maintain continuity for distant families. To date, two patients (P1,2) were lost to follow-up, and one (P3) succumbed to a severe febrile illness at age 5.5. However, the remaining patients (P4, 5, 6, and 7), ranging from 7 months to 8 years, are currently stable with no further crises and satisfactory growth. In one series, despite significant morbidity, long-term survival and even pregnancy are achievable (2).

## Conclusion

In resource-limited settings where advanced laboratory facilities and genetic testing are unavailable, the diagnosis of PHA1 should be considered in any infant, presenting with unexplained fluid loss and electrolyte imbalances once CAH has been excluded. In challenging circumstances, like ours, failure to respond to standard glucocorticoid therapy should immediately raise suspicion of an alternative diagnosis like PHA1. This series helps address the existing gap in published records regarding PHA1 in Sub-Saharan Africa and emphasizes the importance of clinical recognition of the condition in diverse healthcare environments, particularly in low-resource settings where immediate and appropriate intervention can significantly reduce morbidity and mortality.

## Data Availability

The original data presented in the study are included in the article. Further inquiries can be directed to the corresponding authors.
